# Assessment of spino cranial angle of cervical spine sagittal balance system after multi-level anterior cervical discectomy and fusion

**DOI:** 10.1186/s13018-021-02353-1

**Published:** 2021-03-17

**Authors:** Zheng Wang, Zhi-Wei Wang, Xi-Wen Fan, Xian-Da Gao, Wen-Yuan Ding, Da-Long Yang

**Affiliations:** Department of Spinal Surgery, The Third Hospital of Hebei Medical University, 139 Ziqiang Road, Shijiazhuang, 050051 PR China

**Keywords:** Spino cranial angle, Anterior cervical discectomy and fusion, Sagittal balance, Multi-level cervical spondylotic myelopathy, Health-related quality of life scores

## Abstract

**Background:**

To analyze the impact of spino cranial angle (SCA) on alteration of cervical alignment after multi-level anterior cervical discectomy fusion (ACDF) and explore the relationship between SCA and health-related quality of life (HRQOL) scores.

**Material and methods:**

In total, 49 patients following multi-level ACDF for multi-level cervical spondylotic myelopathy (MCSM) with more than 2 years follow-up period were enrolled. Radiographic data including SCA were measured. Receiver operating characteristics (ROC) curve analysis was applied to confirm the optimal cut-off values of SCA for predicting sagittal balance. Patients were divided into two groups on the basis of the cut-off value of preoperative SCA. Correlation coefficients were analyzed between SCA and HRQOL scores.

**Results:**

Optimal cut-off values for predicting sagittal balance was SCA of 88.6°. Patients with higher SCA, no matter preoperatively, postoperatively and at follow-up, got lower T1-Slope (T1s), C2–C7 lordosis angle (CA) and higher △SCA (pre *vs* post: *p* = 0.036, pre *vs* F/U: *p* = 0.022). Simultaneously, pre-SCA, post-SCA, and F/U-SCA in the high SCA group were positively correlated with the pre-NDI, post-NDI, and F/U-NDI scores respectively (pre: *p* < 0.001, post: *p* = 0.015, F/U: *p* = 0.003). However, no correlation was performed in the low SCA group.

**Conclusion:**

An excessive SCA can be considered to cause poorer clinical outcomes at preoperative and better correction after surgery. The SCA could be used as a new reference value to determine sagittal balance parameters of the cervical spine and to assess the quality of life.

## Introduction

Multi-level cervical spondylotic myelopathy (MCSM) refers to a common type of cervical spondylosis with multiple ( ≥ 3) segments, which is often caused by the compression of degenerated facet joints, disks, hypertrophic or ossificated ligamentum flavum and other pathological changes [1]. Multi-level anterior cervical discectomy and fusion (ACDF), as an idiomatic procedure for treating MCSM, can directly achieve the decompression of spinal cord and the correction of kyphosis to a large extent [2]. Analysis of sagittal parameters is critical to understanding cervical spine balance and predicting clinical outcomes. According to previous literatures [3, 4], changes of sagittal parameters may be associated with the quality of life of patients. Recently, Ling, F.P., et al. [5] reported the three most important sagittal balance parameters: T1-Slope (T1s), C2–C7 sagittal vertical axis (cSVA), spino cranial angle (SCA), which will be the focus of future research. SCA refers to a new sagittal parameter, defined as the angle between a line from the sella turcica center and C7 endplate and the C7 plateau line, which must keep in a range (83° ± 9°) under normal conditions. Studies demonstrated a significant correlation between SCA and other important sagittal parameters [6]. Since SCA is a new parameter to evaluate sagittal balance, it has not been extensively investigated.

The purpose of our research is to explore the connection between SCA and health-related quality of life (HRQOL) scores and to explore the impact of ACDF on sagittal parameters, therefore we divided SCA into two categories based on the SCA cut-off value to infer a new reference value to determine cervical sagittal balance and to assess the quality of clinical outcomes.

## Material and methods

### Patient population

Patients with MCSM who had experienced ACDF are retrospectively reviewed from January 2012 to December 2015 at the Department of Spinal Surgery, the Third Hospital of Hebei Medical University. The operative procedure includes at least three levels of intervertebral discectomy, implanting a polyetheretherketone (PEEK) cage filled with softened bone matrix in each intervertebral space and fixing it with a titanium plate. Inclusion criteria are as follows: (1) diagnosed cervical spondylotic myelopathy and magnetic resonance imaging (MRI) examination showed that 3 or more levels were compressed. (2) Relevant data were complete and follow-up period must be more than 24 months. Exclusion criteria are as follows: (1) history of cervical operation; (2) combined with infection, trauma, and tumor; (3) ankylosing spondylitis and rheumatoid arthritis; (4) T1s could not be measured due to shoulder blockage. Imaging information including plain radiography, computed tomography (CT), and MRI of the cervical spine were taken at preoperative, postoperative and follow-up period. We evaluated health-related outcomes preoperatively, postoperatively, and at follow-up, including Japanese Orthopaedic Association (JOA) (score 0–17), recovery rate (RR) (postoperative score-preoperative score)/(17-preoperative score) × 100%.), Neck Disability Index (NDI) (range 0–50), and Quality of life scale (SF-36) (range 0–100).

### Radiographic analysis

**Fig. 1:**
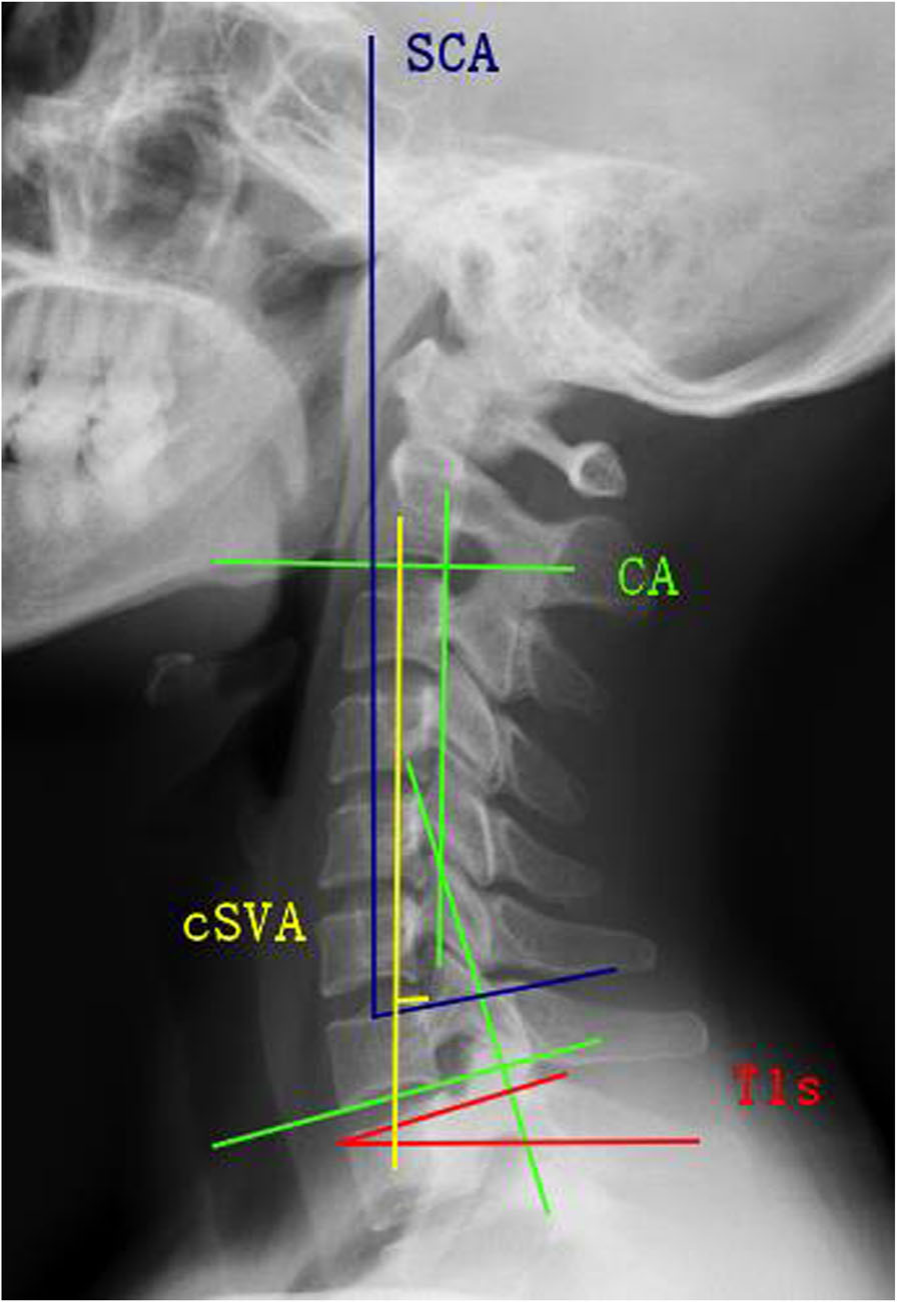
Spino cranial angle (SCA): the angle is defined between the C7 slope and the straight line joining the middle of the C7 end plate and the middle of the sella turcica. T1 slope (T1s): angle between a horizontal line and the superior endplate of T1 or C7. C2–C7 lordosis (CA): angle between the lower plate of C2 and the lower plate of C7. C2–C7 SVA (cSVA): the distance from the posterior, superior corner of C7 to the plumbline from the centroid of C2

Sagittal balance parameters such as SCA, T1s, C2–C7 lordosis (CA), cSVA, T1s minus CA (T1sCA), were measured from lateral X-rays. Imaging parameters were measured (Fig. 1): (1) SCA refers to the angle defined between the C7 slope and the straight line joining the midpoint of the C7 end plate and the midpoint of the sella turcica. (2) T1s refers to the angle between the upper endplate of T1s and a horizontal line. (3) CA refers to the angle between the inferior plate of C2 and the inferior plate of C7. (4) cSVA refers to the horizontal distance from the posterior, superior corner of C7 vertebra to the plumbline from the centroid of C2 vertebra. (5) T1sCA refers to the angle that T1 slope minus C2–C7 lordosis. △SCA, △T1s, △CA, △cSVA, and △T1sCA were all defined as the difference values.

### Statistical analysis

**Fig. 2:**
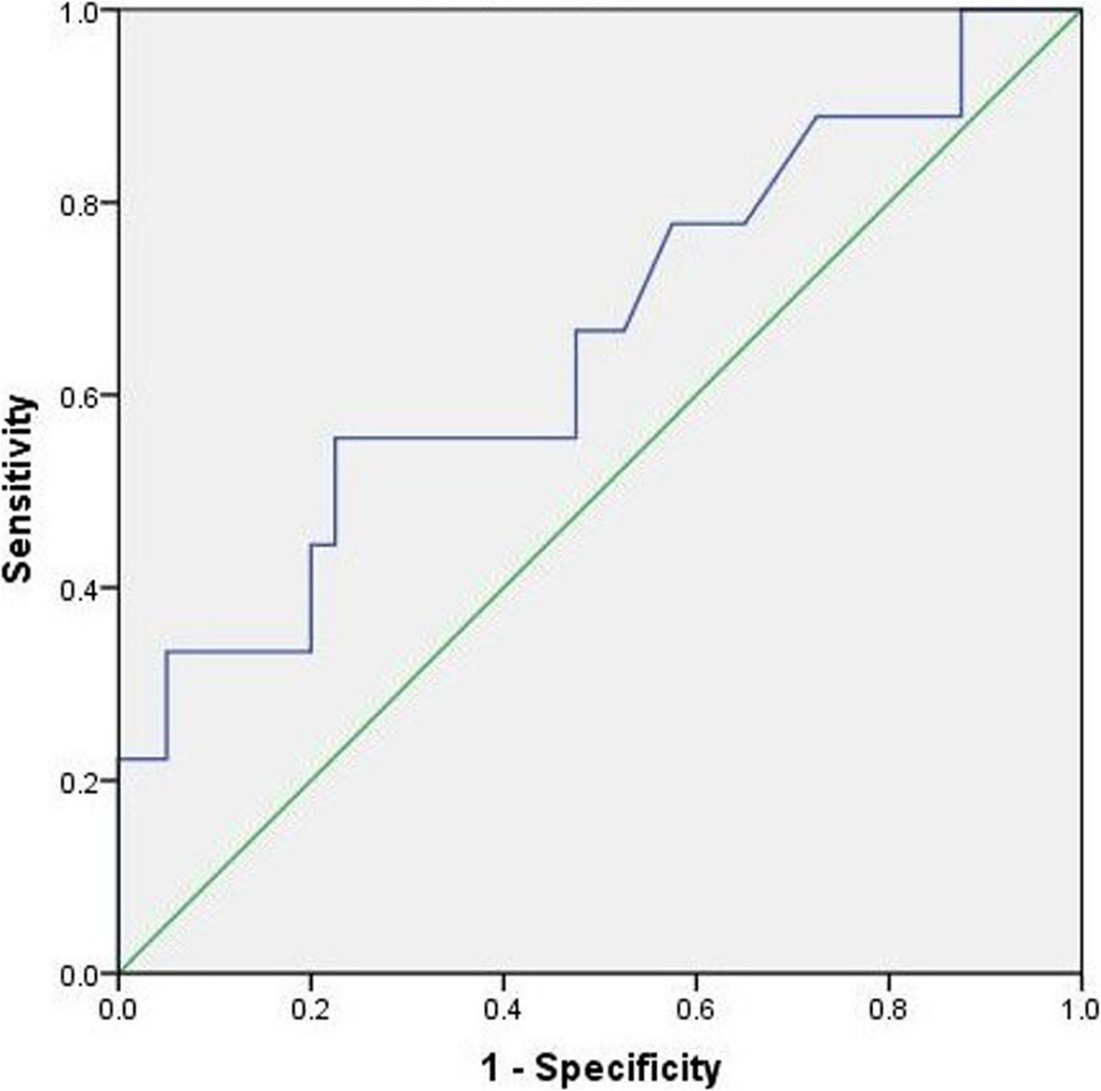
ROC curves of cut-off values of SCA for predicting sagittal balance. AUC results for cut-off values of SCA are 0.660. AUC = area under the curve, ROC=receiver operating characteristics, SCA = spino cranial angle

Receiver operating characteristics (ROC) curve analysis was presented to assess the capacity of preoperative SCA in predicting cervical sagittal balance (Fig. 2). According to the optimal cut-off value of preoperative SCA selected, the patients were then classified into two categories. Normality and homogeneity of variance in the low SCA group and high group were examined and analyzed by Student’s *t* test or Mann–Whitney *U* test. The chi-square test was also applied to evaluate the gender differences and the number of complications in the two groups, respectively. Mean ± standard deviation were calculated by SPSS program (version 22.0; SPSS Inc., Chicago, IL, USA). *p* value < 0.05 was set statistically significant.

## Results

### ROC curve analysis and cut-off value

We used T1sCA to assess cervical alignment (T1sCA ≤ 20°, sagittal balance; > 20°, sagittal imbalance), and the result of ROC curve analysis for cervical sagittal balance showed that preoperative SCA of 88.6° was confirmed as optimal cut-off value. The AUC was 0.660 for cut-off value of SCA (Fig. 2).

### Comparison of demographic data according to preoperative SCA

The demographic data characteristics between the low SCA group (SCA < 88.6°) and the high SCA group (SCA ≥ 88.6°) were summarized in Table 1. All variables, such as age, sex, operation time, operative segments, blood loss, length of hospitalization, and number of complications were not statistically significant between two groups.

**Table 1:** Comparison of patient characteristics

	Low SCA (*n* = 34)	High SCA (*n* = 15)	*p* value
No. of patients Age (year)	34 57.18 ± 6.95	15 55.93 ± 7.51	0.576
Sex (male/female)	17/17	10/5	0.280
Operation time (min)	138.82 ± 31.21	127.33 ± 23.14	0.169
No. of complications	2	3	0.160
Length of hospitalization	6.03 ± 1.77	5.47 ± 1.46	0.199
Operative segments	3.06 ± 0.24	3.07 ± 0.26	0.917
Blood loss	145.29 ± 30.77	143.33 ± 40.65	0.600

### Comparison of sagittal alignment parameters according to preoperative SCA

**Fig. 3:**
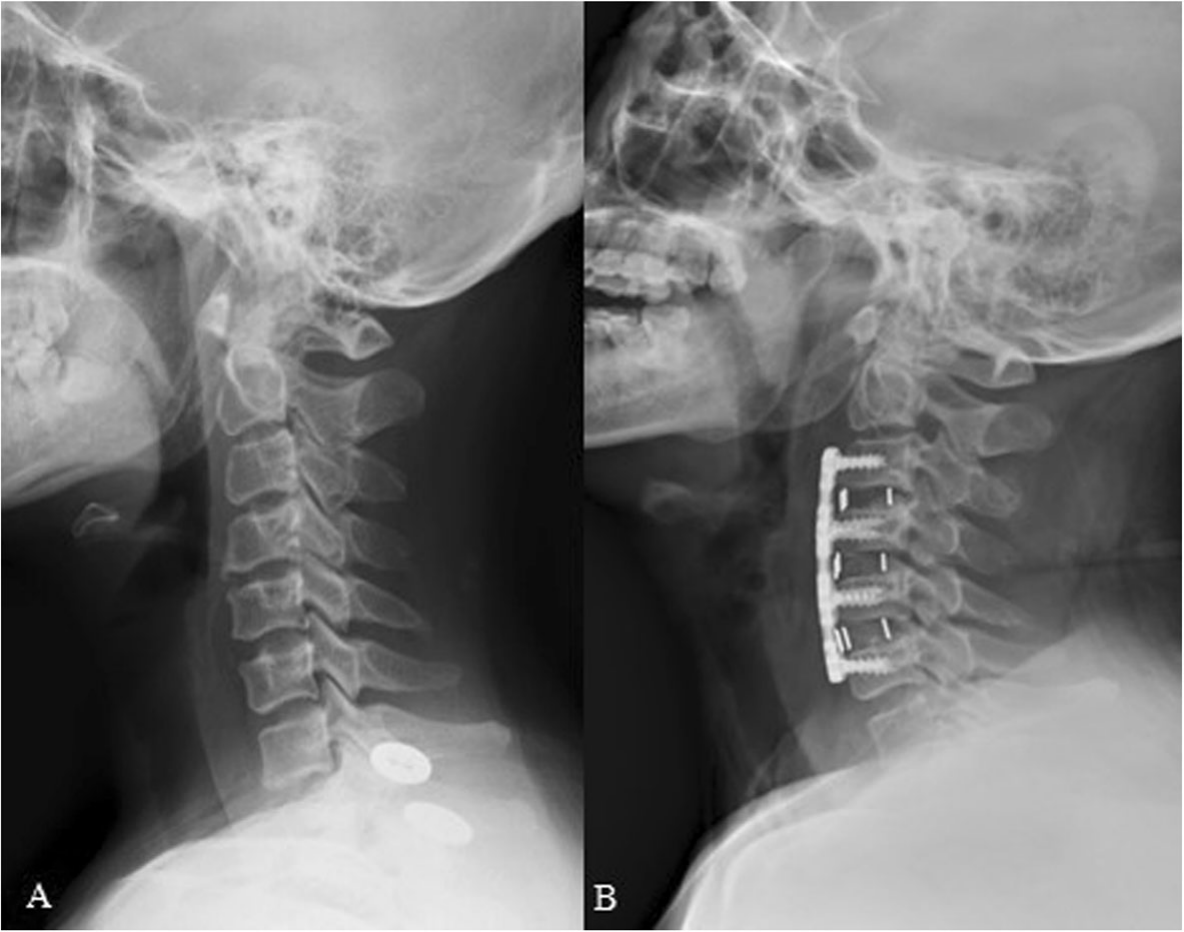
Multi-level ACDF was performed to release the compression. Lateral X-ray of cervical spine was taken in a 56-year-old male patient with MCSM at preoperative (**a**) and 2-year follow-up visit (**b**) and sagittal parameters were corrected appropriately

**Fig. 4:**
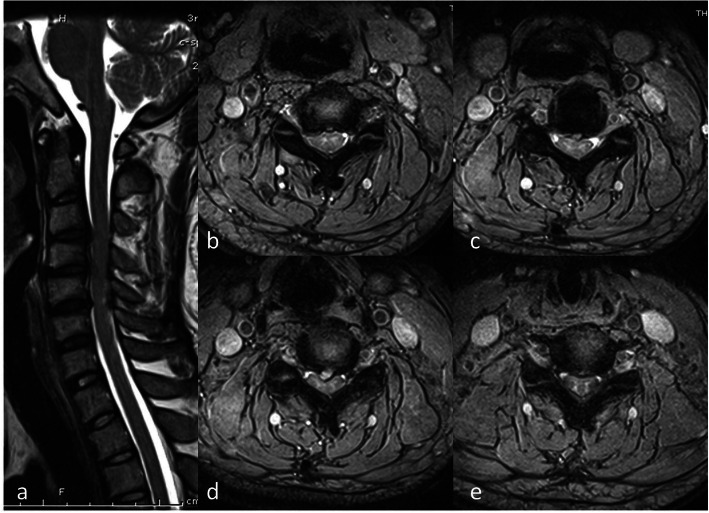
**a** A 56-year-old male patient was diagnosed as MCSM with typical symptoms of superior motor neurons compression. T2-weighted sagittal MRI showed spinal cord compression in C3/4, C4/5, C5/6. **b–e** C3/4, C4/5, C4/5, and C5/6 spinal cord compression on T2-weighted axial MRI

All the surgeries were finished successfully (Figs. 3 and 4). The mean ± standard deviation of pre-SCA, post-SCA, and F/U-SCA were respectively 76.00° ± 6.20°, 73.46° ± 7.91°, and 72.61° ± 7.25° in the low SCA group and 93.85° ± 4.90°, 87.78° ± 3.90°, and 86.73° ± 4.23° in the high SCA group, which all displayed significant differences (*p* < 0.001). Patients with higher SCA preoperatively had more numerical changes than those in the low SCA group at postoperative and follow-up period (post: 2.54° ± 5.59° *vs* 6.07° ± 3.49°, *p* = 0.036, F/U: 3.39° ± 5.68° *vs* 7.11° ± 3.19°, *p* = 0.022). For T1s and CA, significantly statistical difference were found between two groups at preoperative (T1s: *p* < 0.001, CA: *p* < 0.001), postoperative (T1s: *p* = 0.001, CA: *p* = 0.003), and follow-up (T1s: *p* = 0.001, CA: *p* = 0.007). Both △T1s (pre vs post) and △CA (pre vs post) did not show significant differences. However, △CA (pre vs F/U) in the high category was significantly greater than those in the low category (*p* = 0.037), although △CA (pre vs post) did not show the above-mentioned trend. Meanwhile, the postoperative cSVA in the high SCA group (30.06 mm ± 6.03 mm) was significantly larger than that in the low SCA group (24.50 mm ± 7.21 mm) (*p* = 0.012), and there was no significantly statistical difference about T1sCA between two categories for various contrast indicators (Table 2).

**Table 2:** Comparison of cervical sagittal parameters

	Low SCA (*n* = 34)	High SCA (*n* = 15)	*p* value
SCA (°)			
Pre	76.00 ± 6.20	93.85 ± 4.90	< 0.001
Post	73.46 ± 7.91	87.78 ± 3.90	< 0.001
F/U 2y	72.61 ± 7.25	86.73 ± 4.23	< 0.001
△SCA (°)(pre *vs* post)	− 2.54 ± 5.59	− 6.07 ± 3.49	0.036
△SCA (°)(pre *vs* F/U)	− 3.39 ± 5.68	− 7.11 ± 3.19	0.022
Pre *vs* F/U	< 0.001	< 0.001	
T1s (°)			
Pre	30.75 ± 6.42	24.85 ± 3.17	< 0.001
Post	31.29 ± 6.14	26.12 ± 4.01	0.001
F/U 2y	31.25 ± 6.48	26.07 ± 3.30	0.001
△T1s (°)(pre *vs* post)	0.54 ± 3.52	1.27 ± 2.37	0.704
△T1s (°)(pre *vs* F/U)	0.50 ± 3.80	1.23 ± 2.37	0.499
Pre *vs* F/U	0.448	0.065	
CA (°)			
Preoperative	18.29 ± 7.09	9.87 ± 4.90	< 0.001
Post	21.64 ± 8.30	16.05 ± 4.23	0.003
F/U 2y	23.21 ± 8.16	18.35 ± 3.91	0.007
△CA (°)(pre *vs* post)	3.34 ± 4.83	6.18 ± 5.62	0.078
△CA (°)(pre *vs* F/U)	4.91 ± 5.60	8.48 ± 4.77	0.037
Pre *vs* F/U	< 0.001	< 0.001	
cSVA (mm)			
Pre	17.44 ± 5.56	21.15 ± 7.61	0.104
Post	24.50 ± 7.21	30.06 ± 6.03	0.012
F/U 2y	26.79 ± 13.38	27.87 ± 8.43	0.565
△cSVA (mm) (pre *vs* post)	7.06 ± 6.00	8.91 ± 6.45	0.448
△cSVA (mm) (pre *vs* F/U)	9.35 ± 14.18	6.72 ± 7.29	0.397
Pre *vs* F/U	< 0.001	< 0.001	
T1sCA (°)			
Pre	12.46 ± 5.52	14.97 ± 7.06	0.329
Post	9.65 ± 6.27	10.07 ± 5.66	0.826
F/U 2y	8.05 ± 6.27	7.72 ± 5.60	0.863
△T1sCA (°) (pre *vs* post)	− 2.81 ± 5.48	− 4.91 ± 6.74	0.256
△T1sCA (°) (pre *vs* F/U)	− 4.41 ± 6.13	− 7.25 ± 6.09	0.141
Pre *vs* F/U	< 0.001	< 0.001	

*SCA* spino cranial angle, *T1s* T1-Slope, *CA* C2–7 lordosis angle, *cSVA* C2–7 sagittal vertical axis, *T1sCA* T1s minus CA

### Comparison of clinical outcomes according to preoperative SCA

Both two groups were associated with significant enhances in health-related outcomes. Changes in JOA and SF-36 failed to reach significance between two groups, while preoperative NDI in the high SCA category was larger than the low SCA category (23.33 ± 5.70 *vs* 19.32 ± 4.92, *p* = 0.041). △NDI (pre vs post) and △NDI (pre vs F/U) in the high SCA group exceeded than that in the low SCA group (pre *vs* post: *p* = 0.018, pre *vs* F/U: *p* = 0.048) (Table 3).

**Table 3:** Quality of life parameters

	Low SCA (*n* = 34)	High SCA (*n* = 15)	*p* value
JOA			
Pre	10.06 ± 1.56	10.33 ± 1.80	0.877
Post	13.15 ± 1.46	13.20 ± 1.42	0.907
F/U 2y	13.76 ± 1.18	13.73 ± 1.28	0.823
△JOA(pre *vs* post)	3.09 ± 1.55	2.87 ± 1.55	0.647
△JOA(pre *vs* F/U)	3.71 ± 1.59	3.40 ± 2.23	0.912
Pre *vs* F/U	< 0.001	< 0.001	
RR(pre *vs* post)	43.65% ± 23.44%	40.45% ± 20.52%	0.390
RR(pre *vs* F/U)	52.25% ± 19.17%	43.84% ± 35.18%	0.888
NDI			
Pre	19.32 ± 4.92	23.33 ± 5.70	0.041
Post	13.41 ± 3.78	15.53 ± 3.94	0.080
F/U 2y	11.97 ± 3.56	14.13 ± 4.52	0.078
△NDI(pre *vs* post)	− 5.91 ± 2.44	− 7.80 ± 2.54	0.018
△NDI(pre *vs* F/U)	− 7.35 ± 3.05	− 9.20 ± 2.62	0.048
Pre *vs* F/U	< 0.001	< 0.001	
SF-36			
Pre	43.76 ± 6.54	42.80 ± 5.56	0.565
Post	52.12 ± 7.39	52.60 ± 5.83	0.836
F/U 2y	53.47 ± 7.84	53.13 ± 6.40	0.884
△SF-36(pre *vs* post)	8.90 ± 4.39	8.41 ± 4.74	0.420
△SF-36(pre *vs* F/U)	9.88 ± 4.04	8.94 ± 4.56	0.146
Pre *vs* F/U	< 0.001	< 0.001	

*RR* Recovery rate

### Correlation between SCA and other sagittal parameters

There was a close correlation between SCA and T1s (*r* = − 0.685). The difference was significant (*p* < 0.001). We also found a close correlation between SCA and CA (*r* = − 0.831). The difference performed also significant (*p* < 0.001). Similar correlation result was also shown between SCA and T1sCA (*r* = 0.340, *p* = 0.017). Values and the correlation between the different parameters were expressed in Table 4.

**Table 4:** Correlation between SCA and other sagittal parameters

Parameters	Correlation	*p* value	Significance
T1s (°)	− 0.685	< 0.001	S
CA (°)	− 0.831	< 0.001	S
cSVA (mm)	0.219	0.131	NS
T1sCA (°)	0.340	0.017	S

### Correlation between SCA and NDI scores

According to our correlation analysis, in the high SCA group, the pre-SCA, post-SCA, and F/U-SCA were positively correlated with the pre-NDI, post-NDI, and F/U-NDI scores, respectively (pre: *r* = 0.848, *p* < 0.001, post: *r* = 0.611, *p* = 0.015, F/U: *r* = 0.704, *p* = 0.003). However, there was no correlation between SCA and NDI scores in lower SCA group at preoperative (*r* = 0.037, *p* = 0.837), postoperative (*r* = − 0.278, *p* = 0.111), and follow-up period (*r* = − 0.225, *p* = 0.201) (Tables 5 and 6).

**Table 5:** Correlation between SCA and NDI scores in low SCA group

NDI	SCA	Correlation	*p* value
PRE	PRE	0.037	0.837
POST	POST	− 0.278	0.111
F/U	F/U	− 0.225	0.201

**Table 6:** Correlation between SCA and NDI scores in high SCA group

NDI	SCA	Correlation	*p* value
PRE	PRE	0.848	< 0.001
POST	POST	0.611	0.015
F/U	F/U	0.704	0.003

## Discussion

MCSM is a severe threat to patients, which is usually resulted from the extrusion of degenerated facet joints, disks and ossificated ligamentum flavum [1]. It is widely approved multi-level ACDF could remove anterior pathogenic structures directly, which could lead to the extrusion of nerve tissue, such as protruded discs and osteophyte [7], which has been shown to be related to the correction of kyphosis and the maintenance of postoperative lordosis [8, 9]. Nowadays, the influence of multi-level ACDF on sagittal balance has attracted more and more attention [10–14]. Given the important role of sagittal alignment in predicting postoperative clinical effect of MCSM, we are dedicated to studying the relationship between sagittal balance parameters and clinical efficacy increasingly. Recently, as a new sagittal balance parameter, SCA plays an important role in maintaining the balance of the cervical spine, and its postoperative changes will be the focus of future research [5]. In our study, we aimed to discover the influence of ACDF on SCA, which may offer a reference criterion of the cervical sagittal balance and to excavate the relevance between SCA and NDI scores after ACDF for MCSM.

We classified the research subjects into two groups in accordance with cut-off value of SCA. The results showed that no significant difference in variables, such as age, sex, operation time, operative segments, blood loss, length of hospitalization, and number of complications, no matter whether the SCA is high or low. But the results indicated greatly different in the impact of SCA on sagittal parameters. Both T1s and CA are representations of cervical regional to predict sagittal balance. Meanwhile, T1s and CA were both reported to increase significantly after ACDF [15, 16]. Our study demonstrated, compared to the high SCA group, the low SCA group has a higher T1s and CA at preoperative, postoperative, and follow-up period. The T1s between two groups recovered slightly at follow-up visit; however, the F/U-CA between two groups continued the postoperative trend. Meanwhile, in accordance to research [6] concerning SCA to other sagittal parameters, our correlation analysis revealed the pre-SCA was negatively correlated with the pre-T1s (*r* = − 0.685, *p* < 0.001) and pre-CA (*r* = − 0.831, *p* < 0.001), revealing that high T1s may be accompanied with more lordosis and the finding is compatible with previous surveys [9, 17]. In recent years, Kim, T.H., et al. and Oe, S., et al. [18, 19] found that a higher T1s (> 40°) stood for a positive sagittal imbalance and was related to poorer outcomes, which is slightly different from our results, indicating that the low SCA group is accompanied by high T1s (pre: 30.75 ± 6.42, post: 31.29 ± 6.14, F/U: 31.25 ± 6.48), and the moderate increase is considered to reduce the axial symptoms of the neck. It is also in agreement with previous research [20]. At present, the prevailing views have considered that we should pay attention to CA at postoperative and follow-up, which maintains a lower energy expenditure and leads to a positive outcome [21–23]. Interestingly, △CA in the high SCA group significantly exceeded △CA in the low group at follow-up visit (*p* = 0.037); however, the difference did not reach significance at post (*p* = 0.078), revealing that the CA of higher SCA group may be more affected by ACDF over time. As we all know, cSVA is another key parameter for evaluating sagittal balance and predicting quality of life. Previous studies [9, 24] have confirmed that cSVA was negatively correlated with health-related outcomes for multi-level cervical spondylotic myelopathy. Our results indicated that the patients with higher SCA appeared a higher cSVA than patients with lower SCA after surgery, which may manifest that ACDF has greater impact on high SCA group. Unfortunately, on the basis of our correlation analysis, there was no significant correlation between SCA and cSVA (*r* = 0.219, *p* = 0.131). T1sCA (T1sCA = T1s−CA), as a representative of sagittal alignment to evaluate cervical alignment (T1sCA ≤ 20°, sagittal balance; > 20°, sagittal imbalance) [25], is defined to regain horizontal vision after the cervical spine compensated for the T1s by cervical lordosis. We used the results of T1sCA as a criterion for determining balance to classify SCA. Although the regression analysis showed a positive correlation between SCA and T1sCA (*r* = 0.340, *p* = 0.017), unfortunately, after grouping SCA, there was no significant difference between the two groups at various time points (pre: *p* = 0.329, post: *p* = 0.826, F/U: *p* = 0.863). Therefore, it is impossible to further explain the effect of the change in SCA on the compensation effect of T1sCA. The reason for this result may be associated with the cut-off value of SCA.

Compared to the low SCA group, significant changes of SCA were performed in higher SCA group after surgery (*p* = 0.036) and at follow-up period (*p* = 0.022), which may declare that the higher the SCA, the greater the corrective effect of ACDF on sagittal balance. What is interesting in the high SCA group is that the SCA was significantly positively correlated with NDI scores preoperatively (*r* = 0.848, *p* < 0.001), postoperatively (*r* = 0.611, *p* = 0.015), and at follow-up (*r* = 0.704, *p* = 0.003). However, in the low SCA group, there was no significant correlation between SCA and NDI scores preoperatively (*r* = 0.037, *p* = 0.837), postoperatively (*r* = − 0.278, *p* = 0.111), and at follow-up (*r* = − 0.225, *p* = 0.201). We speculate that when SCA is lower than the cut-off value, the change in SCA is not associated with health-related results. Once SCA is higher than the threshold, it will produce worse clinical results as SCA increases. No significant difference in NDI scores at postoperative (*p* = 0.080) and follow-up visit (*p* = 0.078), whether SCA is high or low. However, △NDI in the high SCA group significantly exceeded △NDI in the low SCA group at postoperative (*p* = 0.018) and follow-up visit (*p* = 0.048). Therefore, we believe that the higher the SCA, the better the ameliorate of ACDF on neck pain. The reasons may be the following: when the SCA increases, the muscles and ligaments surrounding the cervical spine are stretched, triggering the threshold of neck pain. And when SCA is below the cut-off value, neck pain can be relieved through posture compensation. Our study showed that once the threshold is exceeded, patients with higher SCA were more likely to bring out worse clinical outcomes, but also more easily relieved after surgery. Therefore, SCA should be focused on when we consider a surgical plan to obtain better treatment effects in dealing with MCSM.

Because the research was retrospective, the study limitations include a slightly small sample, a relatively short follow-up visit and randomized controlled trial.

## Conclusions

This study showed that SCA is negatively correlated with T1s and CA, but positively correlated with T1sCA. An excessive SCA may be considered a bad signal, which is likely to bring out worse clinical outcomes at preoperative but better correction by surgery. The SCA could be used as a new reference value to infer cervical alignment and to assess the quality of life in the future.

## Data Availability

The datasets generated and analyzed during the current study are available from the corresponding author on reasonable request.

## Abbreviations

MCSM: Multi-level cervical spondylotic myelopathy
SCA: Spino cranial angle
T1s: T1-Slope
CA: C2–C7 lordosis angle
cSVA: C2–C7 sagittal vertical axis
T1sCA: T1s minus CA
ACDF: Anterior cervical discectomy and fusion
JOA: Japanese Orthopaedic Association
RR: Recovery rate
NDI: Neck Disability Index
ROC: Receiver operating characteristics
HRQOL: Health-related quality of life
